# Olaparib in combination with irinotecan, cisplatin, and mitomycin C in patients with advanced pancreatic cancer

**DOI:** 10.18632/oncotarget.17237

**Published:** 2017-04-19

**Authors:** Mark Yarchoan, Melinda C. Myzak, Burles A. Johnson, Ana De Jesus-Acosta, Dung T. Le, Elizabeth M. Jaffee, Nilofer S. Azad, Ross C. Donehower, Lei Zheng, Paul E. Oberstein, Robert L. Fine, Daniel A. Laheru, Michael Goggins

**Affiliations:** ^1^ The Sidney Kimmel Comprehensive Cancer Center at Johns Hopkins, Baltimore, MD, USA; ^2^ Columbia University Medical Center, New York, NY, USA

**Keywords:** olaparib, pancreatic cancer, *BRCA2*, irinotecan, cisplatin

## Abstract

**Background:**

Olaparib is an oral inhibitor of polyadenosine 5’-diphosphoribose polymerization (PARP) that has previously shown signs of activity in patients with BRCA mutations and pancreatic ductal adenocarcinoma (PDAC).

**Patients and Methods:**

In this phase 1 dose-escalation trial in patients with unresectable PDAC, we determined the maximum tolerated dose (MTD) of olaparib (tablet formulation) in combination with irinotecan 70 mg/m^2^ on days 1 and 8 and cisplatin 25 mg/m^2^ on days 1 and 8 of a 28-day cycle (olaparib plus IC). We then studied the safety and tolerability of adding mitomycin C 5 mg/m^2^ on day 1 to this regimen (olaparib plus ICM).

**Results:**

18 patients with unresectable PDAC were enrolled. The MTD of olaparib plus IC was olaparib 100 mg twice-daily on days 1 and 8. The addition of mitomycin C to this dose level was not tolerated. Grade ≥3 drug-related adverse events (AEs) were encountered in 16 patients (89%). The most common grade ≥3 drug-related toxicities included neutropenia (89%), lymphopenia (72%), and anemia (22%). Two patients (11%), both of whom had remained on study for more than 12 cycles, developed drug-related myelodysplastic syndrome (MDS). The objective response rate (ORR) for all evaluable patients was 23%. One patient who carried a deleterious germline BRCA2 mutation had a durable clinical response lasting more than four years, but died from complications of treatment-related MDS.

**Conclusions:**

Olaparib had substantial toxicity when combined with IC or ICM in patients with PDAC, and this treatment combination did not have an acceptable risk/benefit profile for further study. However, durable clinical responses were observed in a subset of patients and further clinical investigation of PARP inhibitors in PDAC is warranted.

**Trial registration:**

This clinical trial was registered on ClinicalTrials.gov as NCT01296763.

## INTRODUCTION

Pancreatic ductal adenocarcinoma is an aggressive malignancy and the fourth most common cause of cancer-related deaths in the United States [1, 2]. Only 20-30% of patients with pancreatic ductal adenocarcinoma have resectable disease at diagnosis, and the majority of patients who undergo resection subsequently relapse [3, 4]. Despite the recent development of novel gemcitabine or 5-FU-based combination chemotherapy regimens, the median overall survival for patients with metastatic disease is less than one year [5, 6]. This highlights the continued need for novel and effective therapies for pancreatic ductal adenocarcinoma.

Olaparib (AZD2281, Lynparza™) is an oral inhibitor of polyadenosine 5’-diphosphoribose [poly-(ADP-ribose)] polymerization (PARP), an enzyme essential in the repair of DNA [7–9]. In tumors with defects in homologous DNA repair, PARP inhibition results in the accumulation of single-strand breaks, which are converted during replication to irreparable DNA double-strand breaks and cellular death by apoptosis. Cells with defects in double-strand break repair such as those from BRCA defects are hypersensitive to PARP inhibition, a process called synthetic lethality [10, 11]. Multiple PARP inhibitors have been approved or are in clinical development for ovarian and other cancers. Although PARP inhibitors have clinical activity in cancers without known defects in homologous DNA repair, preclinical and clinical trials have shown higher clinical response rates in patients with tumors harboring such defects [12, 13].

Molecular alterations in homologous DNA repair are known to occur in a subset of pancreatic cancers. Inherited genetic factors account for approximately 5-10% of pancreatic cancer, and *BRCA2* is the most common known germline mutation identified [14]. In addition to BRCA2, other germline mutations that have been implicated in pancreatic ductal adenocarcinoma include *BRCA1, PALB2*, other Fanconi anemia pathway members, and other homologous DNA repair pathways [15, 16]. In one recent cohort study of over 300 patients with pancreatic cancer, pathologic germline mutations in *BRCA2* were identified in 3.3%, *BRCA1* mutations in 1.2%, and *PALB2* in 0% [17]. Somatic mutations affecting genes involved in homologous DNA repair are also identified in a small percentage of pancreatic cancers [18]. In a basket study of single-agent olaparib in patients with a germline *BRCA1/2* mutation, encouraging signs of activity were observed in the subset of patients with advanced pancreatic cancer, with 5 of 23 (21.7%) obtaining an objective response to therapy and multiple patients with stable disease [19]. These results suggested that PARP inhibitors may be effective in the subset of patients with pancreatic ductal adenocarcinoma harboring defects in DNA repair.

PARP inhibitors may also augment the anti-tumor effects of DNA-damaging agents in cancers. Regimens combining PARP inhibitors with cytotoxic agents have synergistic activity in multiple preclinical models [10, 20, 21], but have been limited by unacceptable hematologic toxicity at higher doses [22, 23]. Irinotecan, cisplatin, and mitomycin C (ICM) is an active chemotherapy regimen in pancreatic cancer that has moderate toxicity [24]. In preclinical work (RLF), ICM was highly effective at inducing DNA damage (PARP activity, and apoptosis) in pancreatic cancer cell lines. In an unpublished pilot study of ICM (without olaparib) from our group (RLF), 7 of 10 patients with a known pathologic *BRCA* mutation, and 6 of 20 patients with sporadic pancreatic cancer had an objective response to therapy. The treatment combination was also well tolerated with no grade 3 or 4 toxicities.

In this phase 1 dose-escalation trial, we evaluated the safety and tolerability of olaparib in combination with low-doses of irinotecan and cisplatin (olaparib plus IC), escalating to IC with olaparib plus mitomycin C (olaparib plus ICM), in patients with advanced metastatic pancreatic cancer. We hypothesized that the addition of a PARP inhibitor to low doses of cytotoxic agents would be safe and would potentiate the tumor response to the cytotoxic agents, especially in patients with DNA repair pathway deficiencies.

## RESULTS

### Patients

In total, 18 patients with pancreatic cancer were enrolled and received treatment at Johns Hopkins Kimmel Cancer Center (N=16) and at Columbia University Medical Center (N=2). The clinicopathological characteristics of the patients entered in this study are shown in Table 1. The majority of patients were heavily pretreated, with 13 of 18 (72%) having received 2 or more prior systemic therapies for pancreatic cancer. Two patients (11%) had undergone prior *BRCA* testing and had a known *BRCA2* mutation. None of the other patients enrolled had known or suspected defects in homologous DNA repair.

**Table 1:** Baseline patient characteristics

Characteristic	
**Age, years**	
Median	60
Range	33–76
**Sex, number (%)**	
Male	9 (50%)
Female	9 (50%)
**Race, number (%)**	
White	16 (89%)
Hispanic	1 (6%)
African-American	1 (6%)
**ECOG performance status**	
0	5 (28%)
1	13 (72%)
**Disease stage**	
Locally advanced	1 (6%)
Metastatic	17 (94%)
**Prior therapy**	
Chemotherapy	16 (89%)
Radiotherapy	6 (33%)
Pancreaticoduodenectomy	5 (28%)
**Prior number of chemotherapy regimens**	
0	2 (11%)
1	3 (17%)
2 or more	13 (72%)
**Total number of target lesions**	
1	1 (6%)
2	4 (22%)
3	9 (50%)
>= 4	4 (22%)
**BRCA 1/2 mutation status**	
Positive	2 (11%)
Unknown	16 (89%)
Negative	0

### Sequence of dose levels studied and DLTs

The original dose-escalation strategy is outlined in Table 2.

**Table 2:** Dose-escalation strategy

DoseLevel	Irinotecan	Cisplatin	Olaparib	Mitomycin C
−1	Irinotecan 70 mg/m^2^ on days 1 and 8	Cisplatin 25 mg/m^2^ on days 1 and 8	50mg twice a day on days 1 and 8	None
1	Irinotecan 70 mg/m^2^ on days 1 and 8	Cisplatin 25 mg/m^2^ on days 1 and 8	100 mg twice a day on days 1 and 8	None
2	Irinotecan 70 mg/m^2^ on days 1 and 8	Cisplatin 25 mg/m^2^ on days 1 and 8	100mg twice a day on days 1–3, 8–10	None
3	Irinotecan 70 mg/m^2^ on days 1 and 8	Cisplatin 25 mg/m^2^ on days 1 and 8	200 mg twice a day on days 1–3, 8–10	None
4	Irinotecan 70 mg/m^2^ on days 1 and 8	Cisplatin 25 mg/m^2^ on days 1 and 8	200 mg twice a day on days 1–12	None
5	Irinotecan 70 mg/m^2^ on days 1 and 8	Cisplatin 25 mg/m^2^ on days 1 and 8	MTD from dose level escalation (−1 to 4)	5 mg/m^2^ on day 1

Treatment days are in reference to a 28-day treatment cycle

Dose level 1 (olaparib 100 mg twice-daily on days 1 and 8 plus IC) enrolled six patients. One of the six patients experienced a DLT of grade 4 neutropenia lasting 7 days. Per protocol, the investigators recommended increasing the treatment dose level to level 2.

Dose level 2 (olaparib 100mg twice daily on days 1-3 and 8-10 plus IC) enrolled six patients. One of the patients experienced colonic obstruction at the end of the first cycle of therapy and ultimately died as a result of this complication, but this adverse event was attributed to disease progression rather than drug toxicity and was not considered a DLT. Three of the six patients on dose level 2 experienced a DLT of grade 4 neutropenia lasting greater than 7 days, and the investigators recommended reducing treatment dose to the previous dose level. Therefore, dose levels 3 and 4 were not pursued and olaparib 100 mg twice daily on days 1 and 8 plus IC was selected as the MTD to which mitomycin C was added in dose level 5.

Dose level 5 (olaparib 100 mg twice daily on days 1 and 8 plus ICM) enrolled six patients. Two of these patients experienced a DLT of grade 4 neutropenia lasting greater than 7 days, and one patient experienced a DLT of grade 3 neutropenic fever. Based on these three DLTs, olaparib 100 mg plus ICM was determined to be too toxic for further clinical study.

### Safety and tolerability

All 18 patients were evaluable for safety and tolerability. Four patients (22%) discontinued therapy because of drug toxicity, and ten patients (56%) required dose delays or reductions because of toxicity. Treatment-related adverse events are shown Table 3. Overall, grade ≥3 drug-related adverse events (AEs) were encountered in 16 patients (89%). The most common grade ≥3 drug-related toxicities pertained to hematologic toxicity and included neutropenia (89%), lymphopenia (72%), and anemia (22%).

**Table 3:** Adverse events

Event	Any grade		Grade 3-4	
	No of Patients	%	No of Patients	%
Cardio-renal				
Atrial fibrillation	1	6%		
Elevated creatinine	5	28%	1	6%
Elevated phosphate	2	11%		
Elevated glucose	3	17%		
Hypoalbuminemia	2	11%		
Hypokalemia	3	17%	1	6%
Hypomagnesemia	3	17%		
Hyponatremia	2	11%	1	6%
Hypophosphatemia	2	11%	1	6%
Constitutional				
Dehydration	1	6%		
Fatigue	8	44%	1	6%
Dermatologic				
Alopecia	3	17%		
Gastrointestinal				
Anorexia	3	17%		
Hematemesis	2	11%	1	6%
Hiccups	1	6%	1	6%
Diarrhea	1	6%		
Elevated liver function tests	4	22%	1	6%
Hematochezia	1	6%		
Nausea or vomiting	12	67%	1	6%
HEENT				
Epistaxis	2	11%	2	11%
Hematologic				
Anemia	14	78%	4	22%
Elevated PT/PTT	4	22%	4	22%
Bleeding or bruising	1	6%		
Lymphopenia	15	83%	13	72%
Myelodysplastic Syndrome	2	11%	2	11%
Neutropenia	16	89%	16	89%
Thrombocytopenia	12	67%	1	6%
Infectious				
Sepsis	1	6%		
Febrile neutropenia	4	22%	4	22%
Fever	2	11%		
Neurologic				
Subdural hematoma	1	6%	1	6%

There were 10 drug-related serious adverse events (SAEs) reported. These SAEs included febrile neutropenia (n=3), myelodysplastic syndrome (MDS) (n=2), atrial fibrillation, severe anemia, hematemesis, and a subdural hematoma that occurred in the setting of drug-related thrombocytopenia. Of the three patients who received 12 or more cycles of therapy, two developed MDS. Both patients who developed MDS were in dose level 1 (IC plus olaparib 100 mg twice daily on days 1 and 8) and had experienced an objective clinical response to therapy. Both patients had experienced cytopenias related to therapy that was managed with granulocyte-colony stimulating factor (G-CSF) injections. The first patient who developed MDS had a known *BRCA2* mutation and had obtained no prior systemic therapy for pancreatic cancer. After receiving approximately 2 years of treatment on study with olaparib reduced down to 25 mg bid on day one of each cycle only (in combination with IC), the patient developed worsening cytopenias. A bone marrow aspirate demonstrated dysplastic cell maturation, and chromosomal analysis showed monosomy 7 consistent with therapy-related MDS. The patient received azacitidine treatment for MDS but died from acute myeloid leukemia (AML) almost five years after study initiation. The second patient had received two prior regimens before enrolling on this study, and was on study treatment for approximately one year before discontinuation for disease progression. Approximately six months after discontinuation from study treatment, the patient developed worsening cytopenias and a bone marrow biopsy demonstrated multilineage abnormalities and a borderline increase in blasts consistent with therapy-related MDS.

### Clinical activity

**Figure 1:**
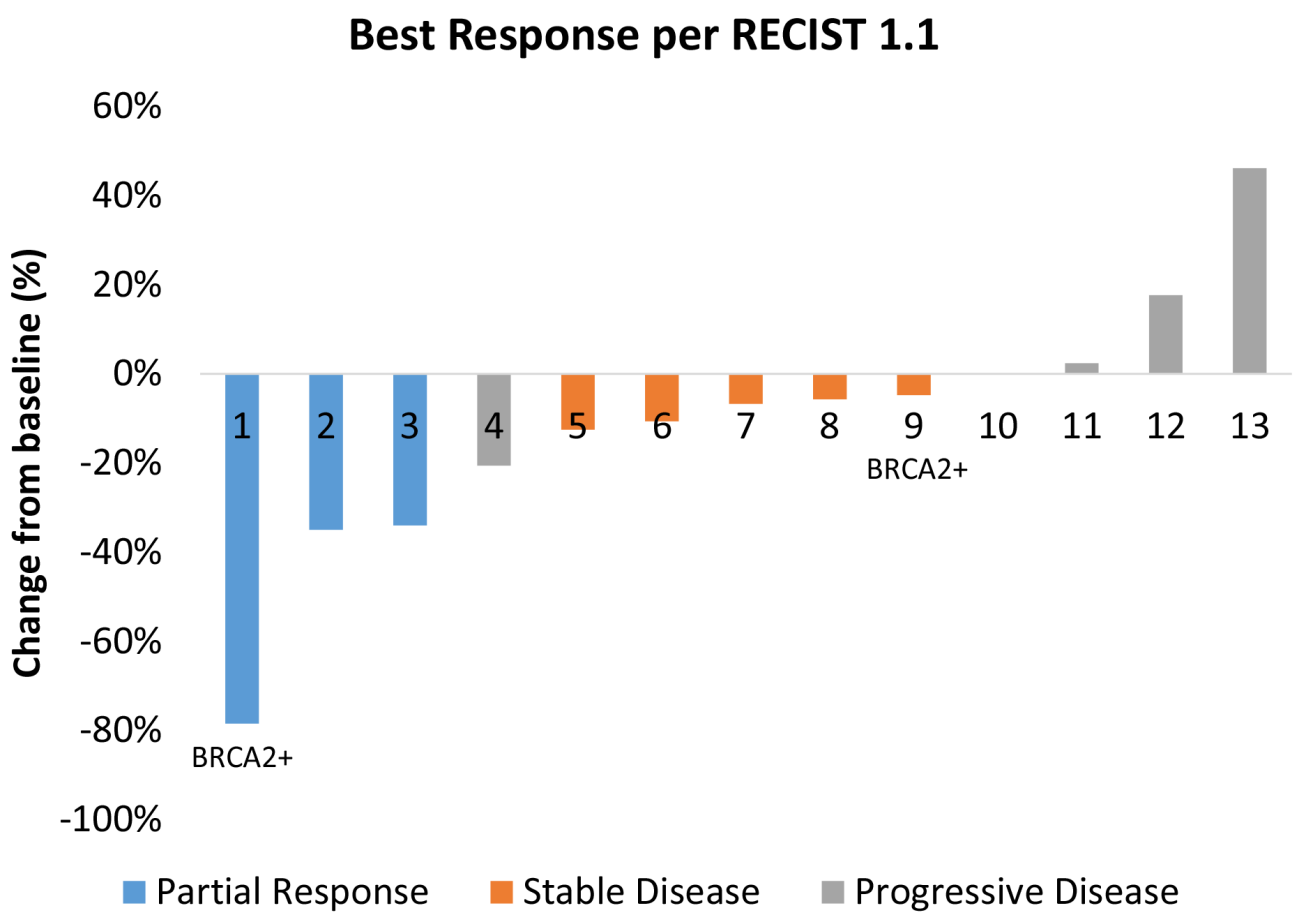
Best tumor response for each evaluable patient The two patients with known BRCA2 mutations are noted. Several patients developed progressive disease because of progression in non-target lesions or the development of new lesions.

Five patients came off study because of toxicity (n=4) or clinical progression (n=1) prior to the first restaging scan. Therefore, 13 patients (72%) were evaluable for clinical activity. The best tumor response for each patient is shown in Figure 1. Three patients experienced a partial response (PR) as a best response to therapy and there were no complete responses (CRs). The ORR for evaluable patients was 23%. Two of the patients who experienced a PR were treated in dose level 1, and one was treated in dose level 5. Five patients (46%) had stable disease (SD) as a best response to therapy. The disease control rate (CR+PR+SD) was 62%. Among the two patients with known *BRCA2* mutations, one experienced a durable PR lasting over four years until death from AML, and the other had stable disease lasting approximately three months as a best response to therapy.

## DISCUSSION

In this study, we found that the combination of olaparib at a dose level of 100 mg twice daily on days 1 and 8 plus ICM had an unacceptable tolerability profile for further study. Treatment with this dose of olaparib plus IC alone was also associated with substantial hematologic toxicity including grade ≥3 drug-related neutropenia, lymphopenia, anemia. Although MDS is a known potential complication of olaparib, the rate of MDS in the present study was higher than we had anticipated, and was higher than previous studies of olaparib monotherapy. In a prior single-arm trial of olaparib monotherapy, MDS/AML was confirmed in 6/298 (2%) of patients [19, 28]. The olaparib dose used in this study was lower than the standard approved monotherapy dose of 300 mg bid daily. In a randomized study of olaparib (300mg BD vs placebo) the incidence of MDS was 1.47% in the olaparib arm and 0.78% in the placebo arm) [29]. In the present study, MDS/AML was observed in 11.1% of patients, albeit in a small sample size. This number may be an underrepresentation of the risk of MDS/AML from the current treatment regimen because many of the patients were on therapy for only a relatively brief time due to intolerance to therapy, and others died of progressive disease before MDS/AML would have developed. The risk of treatment-related MDS/AML is likely related to the duration of drug exposure [19], and therefore it is notable that two out of three patients who remained on study for more than 12 cycles developed MDS/AML in the current study. MDS and AML are clonal processes that arise when hematopoietic progenitor cells acquire specific driver mutations [29]. Although there is no evidence that olaparib alone acts as a mutagenic agent, the combination of chemotherapy-induced DNA damage coupled with an impairment of compensatory DNA repair as a result of PARP inhibition with olaparib may explain the possible MDS signal observed in this study.

PARP inhibitors have previously been successfully combined with chemotherapy in other clinical settings. For example, a recent randomized trial of olaparib plus paclitaxel reportedly only modestly increased rates of neutropenia than paclitaxel monotherapy (30% vs 23%), and no cases of MDS [30]. Olaparib was also successfully combined with gemcitabine in pancreatic cancer without any unmanageable or unexpected toxicities [22]. However, other attempts to combine olaparib with cisplatin-containing regimens have resulted in significant hematologic toxicity [23]. This may be related to the potentiation of cisplatin activity with even intermittent PARP inhibition. Thus, infrequent low-dose olaparib can produce clinically meaningful toxicity when combined with specific chemotherapy regimens. Although PARP inhibitors and DNA damaging agents are a rational drug combination with encouraging signs of activity in preclinical models [10, 20, 21], future clinical trials of combination therapies that include PARP inhibitor should be vigilant about monitoring for hematologic toxicity.

Encouraging evidence of clinical activity was observed in this trial, including a *BRCA2* mutation positive patient who had a remarkable and durable response. However, treatment with ICM alone (without olaparib) also demonstrated clinical benefit in a previous unpublished trial from our group (RLF), and was generally well tolerated. Therefore, the substantial toxicity and modest efficacy observed in present study from the addition of a PARP inhibitor to this prior regimen did not support advancement of this treatment combination into phase 2. Multiple additional studies testing olaparib and other PARP inhibitors to treat pancreatic cancer are ongoing. Use of olaparib monotherapy in the maintenance setting (NCT02184195), or in combination therapy with regimens less likely to cause hematologic toxicity such as irinotecan alone (NCT00576654), may be more successful strategies to incorporate PARP inhibitors into the management of pancreatic cancer. Additionally, the use of PARP inhibitors in pancreatic cancer harboring DNA repair pathway deficiencies may result in higher response rates than were observed in this study, which included patients not selected for such mutations. Limitations of this study include the absence of somatic or germline sequencing data on the majority of participants, and the absence of absence of correlative or pharmacokinetic data for the study agents. Additionally, it is not possible to distinguish the toxicity and efficacy contributions of each component of the studied olaparib plus ICM treatment regimen.

In summary, the combination of olaparib with the DNA damaging regimen IC and ICM was not well tolerated and caused frequent hematologic adverse events. PARP inhibitors continue to be promising therapeutic agents for pancreatic cancer, particularly in the subset of patients with tumors harboring DNA repair pathway deficiencies, and alternative clinical approaches to incorporating these agents into the management of pancreatic cancer should be investigated.

## METHODS

### Eligibility

Patients were eligible for the study if they were 18 years or older with histologic or cytologic confirmation of unresectable pancreatic ductal adenocarcinoma; Eastern Cooperative Oncology Group (ECOG) performance status of ≤2; life expectancy of ≥ 12 weeks; and adequate hematologic, hepatic, and renal function. Measurable disease according to RECIST 1.1 criteria was also required. The presence of a *BRCA* mutation or other cancer DNA repair pathway deficiency was not an eligibility requirement. However, an attempt was made to enrich the study population with patients harboring tumors with DNA repair pathway deficiency who might in theory be most likely to achieve a favorable clinical response to PARP inhibitor-based therapy. This was achieved by providing priority enrollment to patients with known *BRCA1/2* mutations, Jewish individuals (~6% of whom are estimated to carry a germline *BRCA1*/*2* mutation [25]), and patients with familial pancreatic cancer (who are thought to be enriched for defects in homologous DNA repair).

Any number of prior chemotherapy regimens were allowed; however, prior treatment with a PARP inhibitor or more than one drug component of the ICM regimen was not permitted. Additionally, in an attempt to restrict enrollment to patients with adequate hematologic reserve, patients were required to have received ≤12 months of chemotherapy in the metastatic setting. Prior surgery, chemotherapy or other investigational therapies were not permitted within three weeks of the initiation of study treatment. Prior radiation therapy was not permitted within four weeks of the start of study treatment.

### Study design

We used a standard 3 + 3 dose escalation design to determine the maximum tolerated dose (MTD) in this phase 1 dose-escalation trial. The MTD was defined as the highest dose level for which at most 1 out of 6 patients experienced a dose limited toxicity (DLT) during the first cycle of treatment. If 0 of 3 patients had a DLT, the escalation was continued to the next dose level. If 1 of 3 patients had a DLT then 3 more patients were enrolled at that dose. If 1 of 6 patients had a DLT then the escalation was continued. If 2 or more patients treated at a particular dose level had a DLT the dose was reduced to the previous dose level. Subjects continued to receive the specified doses for their cohort until disease progression or toxicity. In the event of drug toxicity, dose reductions of each drug used were based on the clinician's best judgment.

The predetermined dose levels are listed in Table 2. In dose levels 1 to 4, a dose of 70mg/m^2^ of irinotecan and 25mg/m^2^ of cisplatin on days 1 and 8 were studied in combination with escalating doses and frequency of olaparib (100 mg twice daily on days 1 and 8 in dose level 1, up to 200 mg twice daily on days 1-12 in dose level 4) of a 28-day cycle. If patients did not tolerate the starting dose (dose level 1), the frequency of olaparib therapy could be further reduced to once every month (dose level -1). If patients were cisplatin-intolerant or had neuropathy, the protocol allowed for the substitution of carboplatin for cisplatin at a dose of AUC = 3. Once an optimal dose level of olaparib plus IC was obtained (dose levels -1 to 4), then mitomycin C was added to the regimen at a dose of 5 mg/m^2^ on day 1 of each cycle (dose level 5). Granulocyte-colony stimulating factor (G-CSF) was not administered prophylactically unless patients developed persistent neutropenia or febrile neutropenia. In such cases, G-CSF was generally continued prophylactically to limit future episodes of febrile neutropenia.

Participants were enrolled at two academic medical centers, Johns Hopkins Kimmel Cancer Center and Columbia University Medical Center. All patients provided written informed consent for this study. Olaparib was supplied by the Investigational Products Supply (IPS) section of AstraZeneca. The protocol was approved by the Institutional Review Board (IRB) at both study sites, and complied with the International Ethical Guidelines for Biomedical Research Involving Human Subjects and the Declaration of Helsinki. The trial was registered under ClinicalTrials.gov as NCT01296763.

### Response assessments

The primary objective of the study was to determine the MTD. The National Cancer Institute Common Terminology Criteria for Adverse Events (CTCAE) version 4.0 was implemented for adverse event monitoring [26]. A DLT was defined in the protocol as treatment delay of >2 weeks for reasons of toxicity; or thrombocytopenia with platelets <25k for ≥7 days; or grade 4 neutropenia lasting ≥7 days; or grade 3 or 4 febrile neutropenia; or grade ≥3 non-hematological toxicities excluding grade 3 diarrhea, nausea/vomiting, fatigue, lethargy and gamma-glutamyltransferase (GGT) elevation during the first cycle of therapy.

Disease assessments (computed tomography or magnetic resonance imaging) were performed every other cycle of therapy. The objective response rate (ORR) was evaluated according to the Response Evaluation Criteria in Solid Tumors (RECIST), version 1.1 [27]. Upon progression of disease, patients were monitored for long-term adverse events and survival.

### Statistical analysis

The study protocol allowed a maximum of 30 patients to be enrolled in this phase 1 trial to determine the MTD of olaparib in combination with ICM. Descriptive statistics were used to describe the safety and tolerability of the treatment regimen, as no formal statistical comparisons of the data were performed. Patients who developed DLTs requiring discontinuation of the treatment regimen were censored for the efficacy outcomes at the time of discontinuation. Reasonable attempts were made to establish updated responses for patients who withdrew for toxicity prior to scheduled assessments.

## Abbreviations

AEs: adverse events
AML: acute myeloid leukemia
CR: complete response
CTCAE: The National Cancer Institute Common Terminology Criteria for Adverse Events
DLT: dose limited toxicity
ECOG: Eastern Cooperative Oncology Group
GGT: gamma-glutamyltransferase
IC: irinotecan plus cisplatin
ICM: irinotecan plus cisplatin plus mitomycin C
MDS: myelodysplastic syndrome
MTD: maximum tolerated dose
ORR: objective response rate
PARP: polyadenosine 5’-diphosphoribose polymerization
PDAC: pancreatic ductal adenocarcinoma
PR: partial response
RECIST: Response Evaluation Criteria in Solid Tumors
SD: stable disease
